# Method Validation and Evaluation of Safrole Persistence in Cowpea Beans Using Headspace Solid-Phase Microextraction and Gas Chromatography

**DOI:** 10.3390/molecules26226914

**Published:** 2021-11-16

**Authors:** Maria Suely Siqueira Ferraz, Lêda Rita D’Antonino Faroni, Fernanda Fernandes Heleno, Adalberto Hipólito de Sousa, Lucas Henrique Figueiredo Prates, Alessandra Aparecida Zinato Rodrigues

**Affiliations:** 1 Department of Agricultural Engineering, Universidade Federal de Viçosa, Viçosa 36570-900, MG, Brazil; suely.ferraz17@hotmail.com (M.S.S.F.); fernandafhy@yahoo.com.br (F.F.H.); adalberto.sousa@ufac.br (A.H.d.S.); hlucash@gmail.com (L.H.F.P.); alessandra.rodrigues@ufv.br (A.A.Z.R.); 2 Serviço Autônomo de Água e Esgoto, Senador Firmino 36540-000, MG, Brazil; 3 Center of Biology and Natural Sciences, Universidade Federal do Acre, Rio Branco 69920-900, AC, Brazil; 4 Department of Insect Biotechnology in Plant Protection, Justus-Liebig Universität, 35394 Giessen, Germany; 5 Department of Chemistry, Universidade Federal de Viçosa, Viçosa 36570-900, MG, Brazil

**Keywords:** *Vigna unguiculata*, *Piper hispidinervum*, essential oil, stored products, bioinsecticides, sample preparation, HS-SPME-GC/FID

## Abstract

Bioinsecticides are regarded as important alternatives for controlling agricultural pests. However, few studies have determined the persistence of these compounds in stored grains. This study aimed at optimizing and validating a fast and effective method for extraction and quantification of residues of safrole (the main component of *Piper hispidinervum* essential oil) in cowpea beans. It also sought to assess the persistence of this substance in the grains treated by contact and fumigation. The proposed method used headspace solid-phase microextraction (HS-SPME) and gas chromatography with a flame ionization detector (GC/FID). Factors such as temperature, extraction time and type of fiber were assessed to maximize the performance of the extraction technique. The performance of the method was appraised via the parameters selectivity, linearity, limit of detection (LOD), limit of quantification (LOQ), precision, and accuracy. The LOD and LOQ of safrole were 0.0057 and 0.019 μg kg^−1^, respectively and the determination coefficient (R^2^) was >0.99. The relative recovery ranged from 99.26 to 104.85, with a coefficient of variation <15%. The validated method was applied to assess the persistence of safrole residue in grains, where concentrations ranged from 1.095 to 0.052 µg kg^−1^ (contact) and from 2.16 to 0.12 µg kg ^−1^ (fumigation). The levels measured up from the fifth day represented less than 1% of the initial concentration, proving that safrole have low persistence in cowpea beans, thus being safe for bioinsecticide use. Thus, this work is relevant not only for the extraction method developed, but also for the possible use of a natural insecticide in pest management in stored grains.

## 1. Introduction

Cowpea [*Vigna unguiculata* (L.) Walp.], also known as black-eyed pea, belongs to the Fabaceae family and is one of the most cultivated legumes in tropical and subtropical regions, including Asia, Africa, Central and South Americas, the United States, and part of southern Europe [1]. It stands out as an important source of proteins, carbohydrates, vitamins, minerals, and fiber [2].

Many insect pests are linked to losses during the cultivation and storage of agricultural products like cowpea beans [3]. Damage caused by pests during storage—remarkably by *Callosobruchus maculatus* (Fabr., 1775) (Coleoptera: Chrysomelidae)—might compromise yield and the production quality, leading to significant deficits to farmers [4]. Therefore, the primary prevention and control tactic against these attacks has been the use of synthetic insecticides (pyrethroids and organophosphates) and fumigants (such as phosphine, PH_3_) [5,6,7]. Nonetheless, the continuous and indiscriminate use of these products for treating grains has been questioned worldwide, as they are also lethal to non-target organisms [8], lead to resistance development in insect-pest populations [5,9], and potentially pollute the environment [10]. Additionally, they pose concerns about their residual levels in food and the risks to human health [11].

Botanical insecticides stand out among options for insect control for being considered safer and non-associated with the development of pest resistance, consequently presenting a lower contamination risk to the environment and human and animal health [12,13]. Among them are the essential oils (EOs), volatile compounds with an intense odor and, in most cases, a lipophilic constitution, derived from the secondary metabolism of plants [14]. Due to their insecticidal activity, EOs and their chemical constituents are promising alternatives for controlling insect pests in stored grains [15]. The biocidal activity of EOs has been reported against different types of pests and pathogens, such as insects [6,16], mites [17], fungi [18], viruses [19], bacteria [20], nematodes [21], and amoebas [22].

The composition of EOs depends on factors like plant species [23], environmental conditions during cultivation [24], extraction method [16,25] among others. Among other species with potential for pest control, the long pepper (*Piper hispidinervum* C. DC.) is an aromatic plant endemic to the Amazon and has excellent potential for essential oil extraction [26]. Its EO is rich in safrole, which represents about 77.7 to 94.7% of the composition [6,21,22,25,27]. Besides long pepper, safrole is found in EOs from other species of the families Piperaceae [28,29,30], Lauraceae [31], and Myristicaceae [32]. Several studies have evinced the bioinsecticide activity of EOs containing safrole against pest insects of stored grains [6,25,27].

Safrole (4-allyl-1,3-benzodioxole) is a phenylpropanoid used in the synthesis of heliotropin, a fragrance fixative for the cosmetic industry [33], and piperonyl butoxide (PBO), an insecticide synergist employed by the pesticide industry [34]. It is also used as a natural or synthetic flavoring in the food and alcoholic beverage industries [35]. However, studies have revealed the carcinogenic potential of safrole [36,37]. According to the International Agency for Research on Cancer (IARC), safrole is classified in group 2B—possibly carcinogenic to humans [38]. Furthermore, the acute toxicity of safrole to rodents was already established. The lethal doses to kill 50% of populations of rats and mice (LD_50_) were 1950 and 2350 mg kg^−1^ of body weight, respectively [39].

The Codex Alimentarius Commission and the Brazilian Health Regulatory Agency (ANVISA) limit the maximum concentration of safrole in foods and beverages to 1 mg kg^−1^. The exceptions include alcoholic beverages with up to 25% and those with more than 25% alcohol (by volume), whose thresholds are 2 mg kg^−1^ and 5 mg kg^−1^, respectively. The limit is also raised to 15 mg kg^−1^ in foods containing mace and nutmeg [40,41].

The criteria for using *P. hispidinervum* essential oil (PHEO) in stored-grain pest control must consider its post-treatment persistence in the grain mass. Analyses of insecticide persistence are critical to ensuring food security and determining the grace period [14]. Nonetheless, it is remarkable that only a few studies have approached this topic for botanical insecticides applied to stored grains. Therefore, it is necessary to develop analytical methods that quantify lingering volatile compounds of EOs in agricultural products to ensure a safe application.

The solid-phase microextraction technique (SPME) is widely employed in residue analysis, mostly because it performs both extraction and pre-concentration of analytes simultaneously [14,42,43]. The SPME fiber can be used either in the headspace (HS-SPME) or directly immersed in the sample (DI-SPME). However, HS-SPME is more suitable for volatile analytes or highly-complex samples [44]. Some parameters can affect the extraction efficiency, such as the type of fiber coating, the addition of salt to the solution, agitation speed, temperature, time to reach equilibrium in the headspace, fiber exposure time (adsorption of volatile compounds by the fiber), and desorption time (in the chromatograph injector) [45].

Even though several works have investigated the toxicity of EOs to different pests of stored products, it is of great importance to carry out more detailed research on the persistence of these compounds in foodstuff in order to guarantee its safe use. Thus, this study aimed to optimize and validate a method using HS-SPME and gas chromatography with a flame ionization detector (GC/FID) for extracting and analyzing safrole residue in cowpea beans. It also sought to evaluate the persistence of safrole residue in cowpea beans treated with PHEO, either by contact or fumigation, throughout storage.

## 2. Material and Methods

### 2.1. Reagents

The experiment used methanol UV/HPLC grade (Vetec, Duque de Caxias, RJ, Brazil 99.9%), 2000 µg mL^−1^ TraceCERT^®^ safrole solution in methanol (Sigma-Aldrich, Steinheim, Germany, 99.9%), and PHEO extracted via hydrodistillation from *P. hispidinervum* leaves (Federal University of Acre, UFAC, Acre, Brazil). For the optimization and method-validation phase, a stock solution of safrole in methanol was prepared at 500 µg mL^−1^. Then, working solutions were prepared from the stock solution at appropriate concentrations. All solutions were stored in at −18 °C.

### 2.2. Cowpea Bean Characterization

All cowpea used in the experiment (variety *BRS Guariba*) was cultivated pesticide-free in *Codó*, Brazilian state of *Maranhão* (4°27′18″ S latitude, 43°53′09″ W longitude, 43 m altitude). The beans had the following qualitative characteristics: water content = 12.3% (wet basis), germination rate = 98%, and apparent specific mass = 787 kg m^−3^. After harvesting, the beans were stored at −18 °C to avoid contamination with microorganisms until further use. Upon to use, the beans were packed in nylon bags and transported to the Analytical Chemistry Laboratory (LAQUA) at the Federal University of Viçosa (UFV) for further experiments.

### 2.3. Plant Material and Extraction of Essential Oil

*P. hispidinervum* material was collected near the road BR 317, at the kilometer marker 30, *Ramal-Iquiri*, in *Rio Branco*, State of *Acre*, in the Brazilian Amazon Forest (9°58′29″ S latitude, 67°48′36″ W longitude, 153 m altitude). The harvest took place in the morning, and the leaves were sorted from the branches. The leaves were partially dried under ambient conditions and then taken to an oven at 36 °C for drying up until reaching a constant mass. The PHEO extraction was performed in a Clevenger apparatus with a heating mantle (0321A28, Quimis, Diadema, SP, Brazil) and a 5-L volumetric flask. The PHEO was separated from the emulsion by decanting in a separating funnel, using anhydrous sodium sulfate AP (Synth, Diadema, SP, Brazil). The PHEO obtained was stored in an amber bottle at a temperature of 4 ± 1 °C.

### 2.4. P. hispidinervum Essential Oil Composition

The EO compounds were identified and had their relative percentage determined by gas chromatography in the Department of Chemistry (UFV, Viçosa, Brazil). The PHEO was analyzed by gas chromatography combined with mass spectrometry (GC-MS) using the QP2010 system (Shimadzu, Kyoto, Japan). The chromatographic conditions were similar to other applications from our research group [16,46]. Briefly, the following conditions were applied: a fused-silica capillary column (30 m length, 0.25 mm internal diameter), with an RTX^®^-5MS stationary phase (0.25 µm film thickness), and helium as the carrier gas at a flow rate of 1.2 mL min^−1^. The injector was at 220 °C, and the initial temperature of the column was 60 °C. The heating rate was set to increase at 2 °C min^−1^ up to 200 °C, and then at 5 °C min^−1^ up to 250 °C, remaining in this condition for 1 min. The mass spectra were obtained via electron impact at 70 eV, scanning from 29 to 400 (*m*/*z*). The chromatograph operated in full-scan mode and a 1:20 split ratio. The total analysis time was 81 min.

The compounds were identified by matching the resulting mass spectra with those in the NIST library and by visual interpretation. [16,46,47]. Briefly, the compounds were confirmed by the Kovats Index (KI) and comparison with the literature (49451-U, Supelco, Bellefonte, PA, USA, 99.0%). The KI of each compound was calculated based on the retention time of the compounds and the alkanes of the standard alkane solution. The relative percentage of each compound was obtained through the ratio between the area of each peak and the total area of all sample constituents.

### 2.5. Quantification of Absolute Safrole

The safrole in the PHEO and cowpea beans was quantified through GC/FID model 2014 (Shimadzu, Kyoto, Japan), adapting the methods previously published by our research group [16,46,48]. The chromatographic separation was performed in a DB-5 capillary column (Agilent Technologies, Palo Alto, CA, USA), using a stationary phase composed of 5% phenyl and 95% dimethylsiloxane (30 m × 0.25 mm i.d., 0.10 μm film thickness). Nitrogen 99.999% (Air Products, São Paulo, Brazil) was used as the carrier gas in a flow rate of 1.82 mL min^−1^ and a 1:5 split ratio. The temperatures at the injector and detector were set at 220 and 300 °C, respectively. The column was initially at 60 °C, and the temperature increased by 5 °C min^−1^ until reaching 120 °C and hold in this condition for 1 min. The thermal desorption period was 3.0 min, after which the fiber was removed from the injector. Each chromatographic run took 12 min, and all the analyzes were managed by the software GCsolution (Shimadzu, Kyoto, Japan).

Five concentrations of the standard safrole solution (250, 500, 1000, 1500, and 2000 µg mL^−1^) and a PHEO sample of 1000 µg mL^−1^ were diluted in methanol to build the analytical curve and quantify the safrole in the PHEO. These solutions were injected with the aid of the AOC-20i automatic injector and repeated three times. The safrole in the PHEO was determined from the safrole standard analytical curve. The identification of the compound was performed by comparing the retention time of the peak with the retention time of the safrole standard solution in methanol.

### 2.6. HS-SPME Procedure

**Figure 1:**
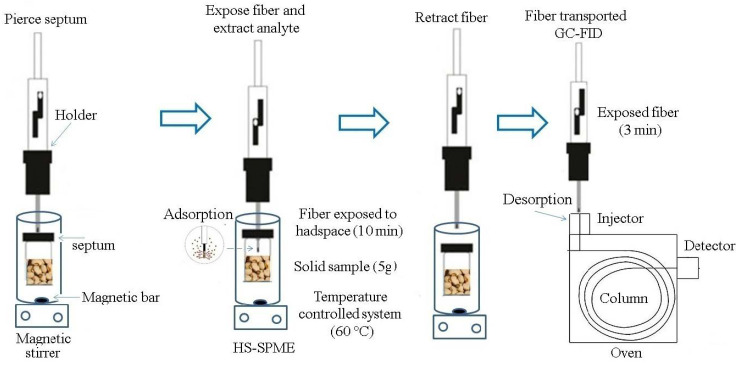
HS-SPME scheme for safrole analysis in cowpea beans samples. Source: Modified from Vilela et al. [48] and Moura et al. [14].

The HS-SPME technique (Figure 1), based on previous publications by our research group [14,46,48], was used for the simultaneous determination and quantification of safrole in cowpea beans. Briefly, the assays were performed using a manual SPME holder (Supelco, Bellefont, PA, USA) equipped with a divinylbenzenecarboxen-polydimethylsiloxane-50/30 μm fiber (DVB/CAR/PDMS-50/30 μm) (Supelco, Bellefont, USA). Samples (5.0 g) were weighed on an analytical balance (Sartorius BP 221 S, Goettingen, Alemanha) in 44 mL amber flasks fitted with a Teflon cap and silicone/PTFE septum. After the fortification with safrole, the samples were homogenized in a vortex (Vortex Mixer CE, Vixar, Korea) for 1 min, and the flasks were allowed to stand for 30 min for solvent evaporation and interaction of the analyte with the matrix. The vials containing the samples were then closed and transferred to a water bath equipped with a magnetic stirrer and set at 60 °C. The fiber (which had been daily conditioned according to the manufacturer’s recommendations) was exposed to the headspace gas phase during the warm-up period (10 min). Following extraction, the fiber was removed from the flask and immediately inserted into a gas chromatography injector, where it remained exposed for 3 min. The analyte was thermally desorbed from the fiber under the carrier gas flow and then led to the chromatographic column.

### 2.7. Optimization of the Method of Safrole Extraction from Cowpea Beans

Safrole was extracted from cowpea beans by HS-SPME-GC/FID. The optimization of this method was divided into two steps: (i) univariate optimization of the fiber to be used, and (ii) multivariate optimization of the extraction time and temperature. At the first stage, four types of fibers produced by Supelco (Bellefort, PA, USA) were tested: 7-μm poly(dimethylsiloxane) (PDMS); 100-μm poly(dimethylsiloxane) (PDMS), 85-μm polyacrylate (PA), and 50/30-μm divinylbenzene/carboxen/poly(dimethylsiloxane) (DVB/CAR/PDMS) fiber. The tests were performed in triplicate, and all fibers were preconditioned as instructed by the manufacturer.

Biopesticide-free cowpea-bean samples (5.0 g) were weighed with an analytical balance inside 44-milliliter amber glass vials. The samples were fortified with safrole at 0.19 µg kg^−1^, using a mixture volume of 2 mL kg^−1^ of cowpea beans, diluted in methanol. Once fortified, they were vortexed for 1 min and let repose for 30 min before the safrole extraction. Next, the flasks with the fortified samples were heated in a water bath at 30 °C with magnetic stirring, and the fibers were exposed for 3 min in the headspace. After the analyte adsorption period, the fibers were collected from the vial and immediately inserted and exposed into the chromatograph injector. The thermic desorption time was 3.0 min, after which the fiber was removed from the injector. In the second optimization stage, a complete 2^2^ factorial design (Table 1) was used to evaluate the behavior of the independent factors: (i) temperature (30 or 60 °C) and (ii) extraction time (3 or 10 min), at the upper (+) and lower (−) levels, in eight trials. These analyzes were performed in duplicate using the fiber that presented the best chromatographic response in the first optimization step.

### 2.8. Validation of the Safrole Extraction and Analysis Method

The validation of the optimized HS-SPME-GC/FID method for extraction and analysis of safrole from cowpea beans considered the following figures of merit: selectivity, linearity, limits of detection (LOD) and quantification (LOQ), accuracy (recovery assays), and precision (repeatability). This method validation was designed following the SANTE/11813/2017 guidelines [49]. In the recovery tests, the biopesticide was added to cowpea beans samples at concentrations equal to one, five, and ten times the LOQ.

### 2.9. Safrole Residue Persistence in Cowpea Beans

Considering the insecticidal potential of safrole, the developed and validated method was used to assess the degradation/persistence rate of its residue in samples of cowpea beans which PHEO was applied with two different methods: contact or fumigation. The beans were treated by contact with PHEO at 208.52 µL kg^−1^ of grains, corresponding to a safrole concentration of 194.77 µg kg^−1^. Another batch of grains were subjected to fumigation with PHEO at 242.59 µL L^−1^ of air, equivalent to a safrole concentration of 226.62 µL L^−1^. These values represent the lethal PHEO dose to kill 95% of a *C. maculatus* population (CL_95_), as determined by preliminary bioassays.

In the treatment by contact, PHEO was sprayed with a gravity-fed double-action airbrush with an internal mixing system (model BC 60, Steula, São Paulo, Brazil). The working pressure used for spraying was 15 psi. In this case, the experimental units consisted of 0.8-L glass flasks (8 cm diameter × 15 cm height), each containing 200 g of cowpea beans. The treatment by fumigation was performed in 300-milliliter glass flasks containing 200 g of cowpea beans per flask. To guarantee no contact of the liquid phase of the PHEO with the grains, PHEO was applied on filter paper (2.5 × 8.0 cm) surrounded by a metallic screen (12.3 × 5.6 × 0.5 cm) with a 4-mm mesh. Organza fabric (15 × 15 cm) was placed upright among the beans. The bottles were closed with a metallic screw lid and sealed with parafilm (PM996, American, Neenah, WI, USA).

After the treatments, the cowpea beans remained exposed for 24 h (contact test) or 96 h (fumigation test)—times determined by previous toxicity bioassays with *C. maculatus*. After that, the grains were transferred to nylon bags (12 × 16 cm) and stored under constant conditions of temperature (25 ± 2 °C) and relative humidity (70 ± 5%). Safrole residue analysis used the validated HS-SPME-GC/FID method, considering storage periods of 5, 10, 26, 60, and 90 days. Safrole persistence was calculated through the analytical curve of the method. All assays were performed in triplicates.

Different mathematical models were tested to fit the degradation of safrole residue. Choosing the best model considered the coefficient of determination (*R^2^*), the estimate of the root-mean-square error (RMSE, the closest to zero), and the significance level (*P* < 0.05).

### 2.10. Statistical Analyses

The data from the chromatographic responses obtained from the safrole solutions in methanol at different concentrations, the analytical curve for safrole extraction, and the persistence curves of safrole residue in cowpea were submitted to analysis of variance (ANOVA) and regression analysis. The chromatographic responses of each SPME fiber exposed to safrole were submitted to ANOVA, and the means were compared by Tukey’s test (*P* > 0.05). The data from safrole extraction from cowpea beans was subjected to ANOVA and surface-response regression analysis, as a function of the extraction time and temperature. The graphs were plotted using Sigmaplot 12.5 (SPSS Inc., Chicago, IL, USA).

## 3. Results and Discussion

### 3.1. Composition of the Essential Oil

The determination of the chemical composition and relative quantification of the compounds in the PHEO were performed via GC-MS. Six components were identified through the chromatographic analysis of PHEO. The main constituents were identified according to their retention index (*RI*) compared to a homologous series of *n-alkanes* and confirmed by comparison of the mass spectrum of the compounds with the NIST-14 spectrometry library.

Safrole was the most abundant compound, accounting for 93.0% of PHEO’s composition, followed by bicyclogermacrene (2.05%), pentadecane (1.60%), spathulenol (1.46%), *p*-cymen-8-ol (1.20%), and (*E*)-caryophyllene (0.69%) (Table 2).

The absolute safrole concentration in PHEO was determined through GC/FID, according to the retention time of this substance. In this case, safrole represented 0.85 mg L^−1^ of the EO constitution. Other studies confirm safrole as the most abundant component of PHEO [6,21,22,27]. The other identified constituents have also been reported in the literature—namely, pentadecane [22], bicyclogermacrene and (*E*)-caryophyllene [6,21,22,27], spathulenol [6,21,27], and *p*-cymen-8-ol [6,27].

### 3.2. Safrole Quantification

The analytical curve of the chromatographic response as a function of the safrole solution concentrations showed significance for the linear model (*y* = a*x* + b) (*P* < 0.0001). The retention time of safrole was 9 min in the GC/FID analysis. The solution of PHEO in methanol at 1.00 mg L^−1^ contained 0.85 mg L^−1^ of safrole, meaning that safrole represented 85% of PHEO composition.

The literature lacks information on the determination of safrole levels by GC/FID. However, analyses carried out via GC-MS also found safrole to be the primary compound in PHEO, with relative concentrations ranging from 77.70 to 94.70% [6,22,25,27].

### 3.3. Optimization of the Safrole Extraction Method

#### 3.3.1. Choosing the Fiber

**Figure 2:**
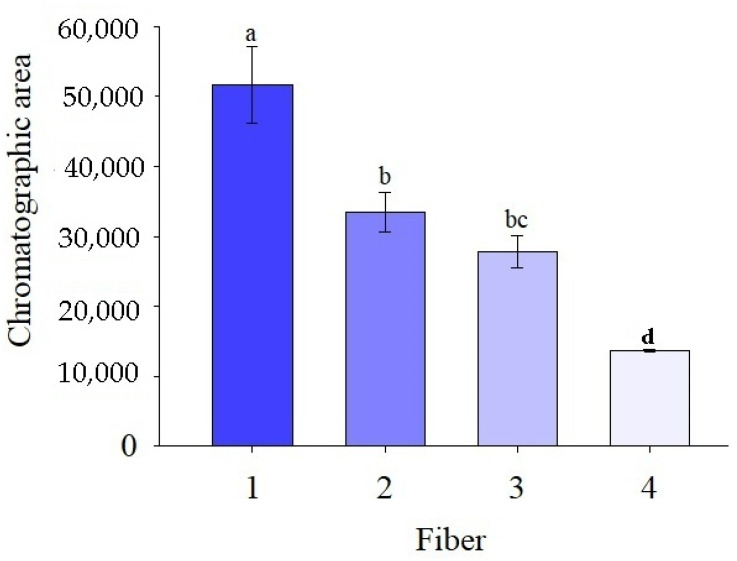
Safrole extraction from cowpea beans by headspace solid-phase microextraction coupled to a gas chromatograph with a flame ionization detector using the fibers: (1) 50/30-μm divinylbenzene/carboxen/polydimethylsiloxane (DVB/CAR/PDMS) StableFlex, (2) 85-µm polyacrylate (PA), (3) 100-µm PDMS, and (4) 7-µm PDMS. Sample mass: 5.0 g; extraction: 30 °C for 3 min (*n* = 3). The same letters are not significantly different by Tukey’s test (*p* < 0.05).

Choosing the fiber for safrole extraction by HS-SPME-GC/FID considered the area of the chromatographic response of each fiber exposed to the compound at 30 °C for 3 min of extraction time. The fibers coated with the different materials herein investigated presented distinct behavior, as statistically attested by Tukey’s test (*P* < 0.05). The 50/30-µm DVB/CAR/PDMS StableFlex fiber differed from both 85-µm polyacrylate (PA), 100-µm fused-silica PDMS and 7-µm fused-silica PDMS ones (Figure 2). The 50/30-µm DVB/CAR/PDMS StableFlex fiber differed from both 100-µm fused-silica PDMS and 7-µm fused-silica PDMS ones; whereas the 85-µm fused-silica PA fiber only differed from the 7-µm fused-silica PDMS (Figure 2). Ultimately, the fiber of choice was the 50/30-μm StableFlex coated with DVB/CAR/PDMS, which delivered a response with a larger chromatographic area, indicating better safrole extraction. Polarity, molecular mass, and size of the analytes are criteria that also must be accounted for determining the most appropriate fiber for HS-SPME [42].

Xiao et al. [45] assessed the performance of four fibers with different coatings for extracting volatile compounds from cherry wine—namely, 50/30-μm DVB/CAR/PDMS StableFlex, 65-μm PDMS/DVB, 75-μm CAR/PDMS, and 100-μm PDMS fibers. According to that study, the 50/30-μm DVB/CAR/PDMS StableFlex fiber had the best performance for extracting 3-methyl-butanol, ethyl hexanoate, hexyl acetate, ethyl lactate, and 4-ethylguaiacol. This fiber also produced the best response for extracting aromatic compounds from cocoa by HS-SPME-GC/FID compared to fibers coated with PDMS, PDMS/DVB, and CAR/PDMS [50]. These findings support the results from the present study.

Fibers coated with PDMS have long-term stability and performance regarding chromatography, extraction characteristics, and analyte recovery. In addition, they are durable and typically maintain good functioning for up to 100 analytical cycles. As these fibers are non-polar, the manufacturers suggest them for extracting non-polar analytes, such as aromatic and volatile compounds from EOs. As for mixed fibers, whose coating contains DVB or CAR, their retention capacity is raised due to a mutual potentiating effect on the extraction and distribution of the stationary phase. Double-coated DVB/CAR/PDMS fibers have a layer of PDMS-DVB over another of CAR-PDMS. They are also recommended for flavor and odor extraction (either volatile or semi-volatile) in foodstuff [50].

#### 3.3.2. Optimal Conditions of Temperature and Extraction Time

**Figure 3:**
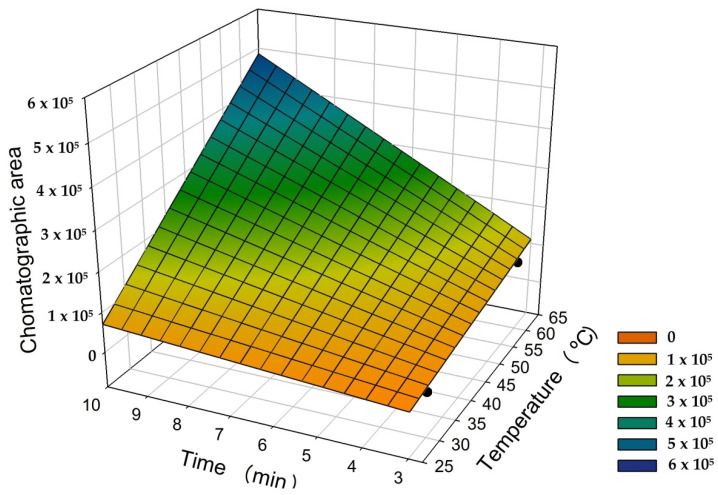
Optimization of safrole extraction from cowpea beans by the headspace solid-phase microextraction method coupled to a gas chromatograph with a flame ionization detector, as a function of temperature and extraction time.

The best experimental conditions were obtained at 60 °C and 10 min of extraction time. The largest chromatographic area by the GC-FID method were obtained with these settings (Figure 3). The response surface regression analysis exhibited linear behavior for both parameters (*z* = 1172.89*xy* − 1356.22*x* − 22662.76*y* + 38058.24; where *x* is the temperature, and *y* is the extraction time) (*R^2^* = 0.996; *P* < 0.05).

The SPME technique involves a multiphase equilibrium process. In general, extraction time and temperature are among the most critical factors affecting SPME, thus being the usual variables chosen for investigation and optimization [51]. Equilibrium time in the headspace is related to the extraction temperature, fiber coating thickness, and analyte diffusion coefficients [52]. The analyte is released from the matrix more efficiently when exposed to high temperatures. This phenomenon occurs due to the reaction kinetics, as the molecular kinetic energy of the analyte is directly proportional to the temperature rising [51].

Jeong et al. [39] used HS-SPME-GC/FID with a 100-µm PDMS fiber to remove volatile monoterpenes from *Mentha piperita* L. (Lamiaceae) extract. The optimal extraction conditions for these compounds were found at 64 °C and 28.5 min extraction time. The optimal temperature determined by the authors is close to the temperature found in the present study. However, their exposure time was longer, probably because of the fiber coating they employed (100-µm PDMS), which delivered a low performance in this research.

Results found by Ducki et al. [50] corroborate the present investigation. Likewise, they observed that the most satisfactory settings for extracting aromatic compounds from cocoa were 60 °C and 15 min extraction time, employing a 50/30-μm DVB/CAR/PDMS StableFlex fiber.

### 3.4. Validation of the Method of Extraction and Analysis

**Figure 4:**
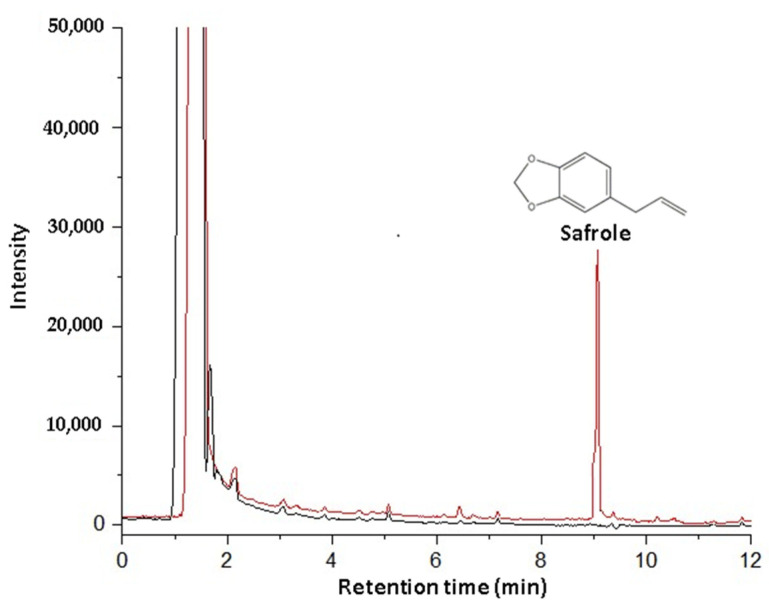
In red, chromatogram obtained from the analysis of beans fortified with safrole (t_R_ = 9.0 min) and black, chromatogram of white matrix + methanol after extraction and analysis by HS-SPME-GC/FID, (*n* = 3).

The HS-SPME method for safrole determination in cowpea beans was validated, and the results fulfilled the guidelines by SANTE/11813/2017 [49]. Untreated bean samples (blank) and samples treated with safrole were exposed to the extraction method and analysis to assess selectivity. Then, the resulting chromatograms were compared (Figure 4). No interference was noticeable in the retention time of the compound of interest, therefore supporting the selectivity of the technique.

The LOD of the method was determined considering three times the signal-noise (*S*/*N* = 3) baseline of the safrole-free cowpea-bean samples (blank) in a GC/FID analysis. In turn, the LOQ was calculated assuming a signal at least ten times higher than the noise (*S*/*N* = 10). The resulting LOD and LOQ values (0.0057 µg kg^−1^ and 0.019 µg kg^−1^ of cowpea beans, respectively) were considerably lower than the maximum concentration allowed for safrole usage in the food industry. The LOD represents the lowest concentration of an analyte in a sample that can be detected, but not necessarily quantified, under the stated conditions of the test. The LOQ is the lowest concentration of an analyte in a sample that can be determined with acceptable precision and accuracy under the testing conditions [49,53].

The response linearity of the method was determined by assessing cowpea-bean samples fortified at seven different concentrations of safrole (1 × LOQ, 5 × LOQ, 10 × LOQ, 50 × LOQ, 100 × LOQ, 150 × LOQ, and 200 × LOQ). After fortification, the samples (5.0 g) were submitted to the extraction and analysis method in triplicate. The resulting data were plotted in an analytical curve with seven points (*n* = 3), relating the analyte areas to the corresponding concentrations. The analytical curve for safrole extraction from cowpea beans was significant for the linear model (*y* = a*x* + b) (*P* < 0.0001). The linearity of the method ranged from 0.019 to 3.80 µg kg^−1^ of cowpea beans, with a coefficient of determination above 0.99 (*R^2^* = 0.9990). The analytical curve indicates the relationship between the theoretical analyte concentration in the sample and the chromatographic area [54].

The precision of the method was investigated via repeatability assays. Samples of cowpea beans were fortified at three levels of concentration of safrole (1 × LOQ, 5 × LOQ, and 10 × LOQ) in six replicates. The repeatability was determined by the coefficient of variation (CV) of the concentrations obtained experimentally by HS-SPME-GC/FID. These procedures were carried out on the same day, with the same equipment, and by the same analyst.

Precision and accuracy were appraised at the levels of 0.019, 0.095, and 0.190 µg kg^−1^ of cowpea beans (Table 3). The average recovery ranged from 99.26 to 104.85% at these three concentrations, thus within the acceptable standards for chromatographic methods (70–120%) [49]. The HS-SPME-GC/FID method showed recovery rates close to 100% and good precision, with a CV equal to or less than 14.82%. In residue analysis, coefficients of variation of up to 20% are accepted [49].

HS-SPME-GC/FID has advantages over other methods because it reduces or eliminates the need for organic solvents. Besides, it makes it possible to extract and concentrate the analyte simultaneously, thefore not requiring additional procedures for sample cleanup [42,43,51].

### 3.5. Application of (HS-SPME) Method for Evaluating the Persistence of Safrole in Cowpea Beans

**Figure 5:**
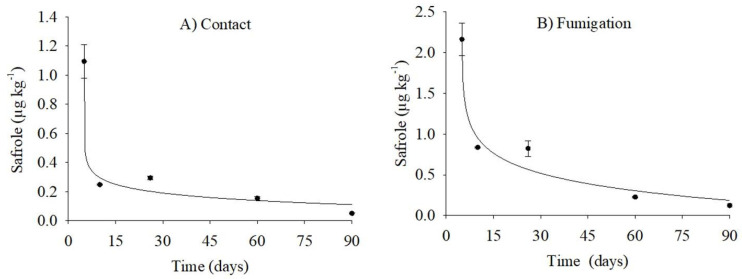
Persistence of safrole residue in cowpea beans treated with *Piper hispidinervum* essential oil, either by contact (**A**) or fumigation (**B**), throughout storage. Sample mass: 5.0 g; extraction: 60 °C for 10 min (*n* = 3).

Different mathematical models were tested to explain the safrole-residue degradation in cowpea beans treated with PHEO, both by contact and fumigation (Table 4). For contact-treated grains, the best fit was the exponential model (y = aе^-bx^ + c) (Figure 5a), with *R^2^* = 0.96, RMSE = 0.077, and *P* = 0.043. On the other hand, the best fit for fumigated grains was the logarithmic model (*y* = *y_0_* − a ln (*x* − *x_0_*)) (Figure 5b), with *R^2^* = 0.97, RMSE = 0.14, and *P* = 0.034.

Safrole residue was evaluated throughout storage. The concentrations detected in the contact-treated grains were 1.095 µg kg^−1^ and 0.052 µg kg^−1^, after 5 and 90 days, respectively. For fumigated grains, the safrole residue levels were 2.16 µg kg^−1^ and 0.12 µg kg^−1^, after 5 and 90 days. Overall, by the fifth day of storage, the safrole levels in the grains was reduced by 99%. The safrole concentrations found in the cowpea-bean after treatments are lower than the limits set for beverages and foodstuff by Codex Alimentarius and Anvisa [40,41]. Nonetheless, these dose levels proved to control pests in stored cowpea beans, such as *C. maculatus*.

The effect of residual PHEO on *C. maculatus* and *Sitophilus zeamais* Mots. (Coleoptera: Curculionidae) was investigated in cowpea beans and corn grains stored for up to 120 days by Pereira et al. [55] and Coitinho et al. [25], respectively. The toxicity of PHEO was the highest right after grain contamination, with values ranging from 100 to 93.8% mortality. After 30 days of storage, a low residual effect was noticed, with less than 17.5% mortality. In the present study, 99% of PHEO degradation occurred after 5 days of storage, which may explain the low toxicity found by Pereira et al. [55] and Coitinho et al. [25] after 30 days of storage.

Studies showed that botanical insecticides have low persistence in stored products [25,55,56]. Allicin, the major compound in the EO of *Allium sativum* L (Amaryllidaceae), was monitored during and after fumigation of wheat, followed by aeration of the grains [56]. The concentration of allicin used in the procedure was 615 μL L^−1^ of air, but the compound level decreased as the fumigation and aeration time increased, resulting in an exponential behavior. The concentration dropped by 98.99% after one day after the procedure, and by day 16, this reduction reached 99.99%.

The chemical instability of their constituents explains the degradation of EOs. Oxidation, isomerization, cyclization, and dehydrogenation reactions triggered by enzymes or other molecules (such as oxygen) are common. Temperature, light, and availability of atmospheric oxygen also influence the stability of EOs [57].

## 4. Conclusions

The HS-SPME-GC/FID method was optimized for extracting safrole from PHEO-treated cowpea beans. The optimal conditions were 60 ºC and 10 min extraction time, using a 50/30-μm DVB/CAR/PDMS StableFlex fiber. The optimized method showed good linearity (within the tested range), repeatability, and suitable LOD and LOQ. During the storage of cowpea beans treated with PHEO by contact, safrole concentrations varied from 1.095 to 0.052 µg kg^−1^. When the compound was applied by fumigation, the levels ranged between 2.16 and 0.12 µg kg^−1^.

PHEO had low persistence in cowpea beans treated by either fumigation or contact. The safrole residue detected after 5 days of storage represented less than 1% of the initial concentration.

Based on our current results and the insecticidal potential already reported in the literature, it is possible to conclude that PHEO shows potential to preserve the quality of stored cowpea beans by inhibiting the development of this insect-pest species. Moreover, safrole has low persistence in the grains, suggesting that PHEO is a safe option that might be also suitable for treating similar food products. Additionally, the optimized HS-SPME-CG/FID method can be successfully used to determine the presence of safrole in cowpea beans.

## Figures and Tables

**Table 1:** Complete 2^2^-factorial design for optimization of the safrole extraction conditions from cowpea beans.

Experiments	Time (min) ^a^		Temperature (°C) ^b^	
	Real	Codified	Real	Codified
1	3	−	30	−
2	3	−	60	+
3	10	+	30	−
4	10	+	60	+
5	3	−	30	−
6	3	−	60	+
7	10	+	30	−
8	10	+	60	+

^a^ Exposure time of the fiber; ^b^ temperature (°C) of the water bath with magnetic stirring. The data correspond to the (−) lower value and (+) upper value levels of factorial design.

**Table 2:** Chemical composition of *Piper hispidinervum* essential oil and relative concentrations of the compounds identified by gas chromatography analysis coupled with mass spectrometry.

Component	*RI*^a^ Literature	*RI*^b^ Calculated	Relative %
*p*-Cymen-8-ol	1179	1184	1.20
Safrole	1285	1292	93.00
(*E*)-Caryophyllene	1417	1415	0.69
Bicyclogermacrene	1500	1493	2.05
Pentadecan	1500	1498	1.60
Spatulenol	1577	1573	1.46

^a^ Relative retention index taken from (Adams, 2007) and/or NIST 14 and ^b^ Retention index experimentally determined using homologous series of C_7_–C_30_ alkanes (Kovats index) [47].

**Table 3:** Accuracy and precision of the headspace solid-phase microextraction method coupled with a gas chromatograph with flame ionization detector for extraction of safrole from cowpea beans.

Theoretical Concentration	Nominal Concentration ± ^1^SE	Accuracy (n = 6)	Precision
(µg kg^−1^)	(µg kg^−1^)	Recovery (%)	^2^CV (%)
0.019	0.018 ± 0.0010	99.26	12.76
0.095	0.094 ± 0.0057	100.48	14.82
0.190	0.196 ± 0.012	104.85	14.61

^1^SE = standard error; ^2^CV = coefficient of variation.

**Table 4:** Mathematical-model fitting for safrole residues in cowpea beans treated with *Piper hispidinervum* essential oil, either by contact (a) or fumigation (b), throughout storage.

Treatment	Model	Equation	*R* ^2 a^	RMSE ^b^	*P*
Contact	Linear	*y* = − 0.0079 *x* + 0.67	0.46	0.27	0.21
	Quadratic	*y* = 0.0002*x*^2^ − 0.025*x* + 0.86	0.58	0.25	0.42
	Exponential	*y* = 10.54еxp^−0.4861*x*^ + 0.17	0.98	0.08	0.043
	Potential	*y* = 18.06(1 + *x*) ^−1.5842^	0.88	0.13	0.018
	Logarithmic	*y* = − 0.065ln (*x* − 5.0000) + 0.40	0.98	0.49	0.021
Fumigation	Linear	*y* = − 0.018*x* + 1.5	0.64	0.43	0.10
	Quadratic	*y* = 0.0004*x*^2^ − 0.052*x* + 1.9	0.76	0.38	0.24
	Exponential	*y* = 6.53еxp^−0.2606*x*^ + 0.38	0.89	0.24	0.11
	Potential	*y* = 11.16(1 + *x*) ^−0.95^	0.92	0.21	0.011
	Logarithmic	*y* = − 0.27ln (*x* − 4.94) + 1.4	0.97	0.13	0.034

^a^ Coefficient of determination and ^b^ Root-mean-square error (*n* = 3).

## Data Availability

Not applicable.

## Sample Availability

Samples of the compounds are not available from the authors.
